# Author Correction: C16orf72/HAPSTR1/TAPR1 functions with BRCA1/Senataxin to modulate replication-associated R-loops and confer resistance to PARP disruption

**DOI:** 10.1038/s41467-023-43353-5

**Published:** 2023-11-27

**Authors:** Abhishek Bharadwaj Sharma, Muhammad Khairul Ramlee, Joel Kosmin, Martin R. Higgs, Amy Wolstenholme, George E. Ronson, Dylan Jones, Daniel Ebner, Noor Shamkhi, David Sims, Paul W. G. Wijnhoven, Josep V. Forment, Ian Gibbs-Seymour, Nicholas D. Lakin

**Affiliations:** 1 Department of Biochemistry, University of Oxford, South Parks Road, Oxford, UK; 2 Institute of Cancer and Genomic Sciences, University of Birmingham, Edgbaston, Birmingham UK; 3 Target Discovery Institute, Nuffield Department of Medicine, University of Oxford, Oxford, UK; 4 Weatherall Institute of Molecular Medicine, University of Oxford, John Radcliffe Hospital, Oxford, UK; 5 Early Oncology R&D, AstraZeneca, 1 Francis Crick Avenue, Cambridge Biomedical Campus, Cambridge, CB2 0AA UK

**Keywords:** DNA damage and repair, DNA replication

Correction to: *Nature Communications* 10.1038/s41467-023-40779-9, published online 17 August 2023

The original version of this Article contained an error in the spelling of the author Josep V. Forment, which was incorrectly given as Josep Forment. This has now been corrected in both the PDF and HTML versions of the Article.

