# Supplementary material for: C16orf72/HAPSTR1/TAPR1 functions with BRCA1/Senataxin to modulate replication-associated R-loops and confer resistance to PARP disruption
Source: Nat Commun. 2023 Aug 17;14:5003. doi: 10.1038/s41467-023-40779-9 (PMC10435583; doi:10.1038/s41467-023-40779-9)
Supplement: Supplementary file 1 — Supplementary Information [file 41467_2023_40779_MOESM1_ESM.pdf]

a

```
U2OS WT          ATGGAGGAGCGGAAGGAGGAGGGCGAGGCCGAGATCCAGGAGCACGGGCCCGAGCACTGGTTCTCCAAGTGGGAGCGGCA
c16orf72Δ.2      ATGGAGGAGCGGAAGGAGGAGGGCGAGGCCGAGATCCAGGAAGCACGGGCCCGAGCACTGGTTCTCCAAGTGGGAGCGGCA (Allele 1)
                 ATGGAGGAGCGGAAGGAGGAGGGCGAGGCCGAGATCCAGGA---------(deletion of 56 bp)-----------  (Allele 2)
c16orf72Δ.3      ATGGAGGAGCGGAAGGAGGAGGGCGAGGCCGAGATCCAGGA---------(deletion of 56 bp)-----------

U2OS WT          GTGCCTGGCCGAGGCCGAACAGGACGAGCAGCTGCCCCCCGAGCTGCAGGAGGAGGCGGCGGCCGCCGCGCAGCCCGAGC
c16orf72Δ.2      GTGCCTGGCCGAGGCCGAACAGGACGAGCAGCTGCCCCCCGAGCTGCAGGAGGAGGCGGCGGCCGCCGCGCAGCCCGAGC  (Allele 1)
                 -----------------AACAGGACGAGCAGCTGCCCCCCGAGCTGCAGGAGGAGGCGGCGGCCGCCGCGCAGCCCGAGC  (Allele 2)
c16orf72Δ.3      -----------------AACAGGACGAGCAGCTGCCCCCCGAGCTGCAGGAGGAGGCGGCGGCCGCCGCGCAGCCCGAGC
```

b

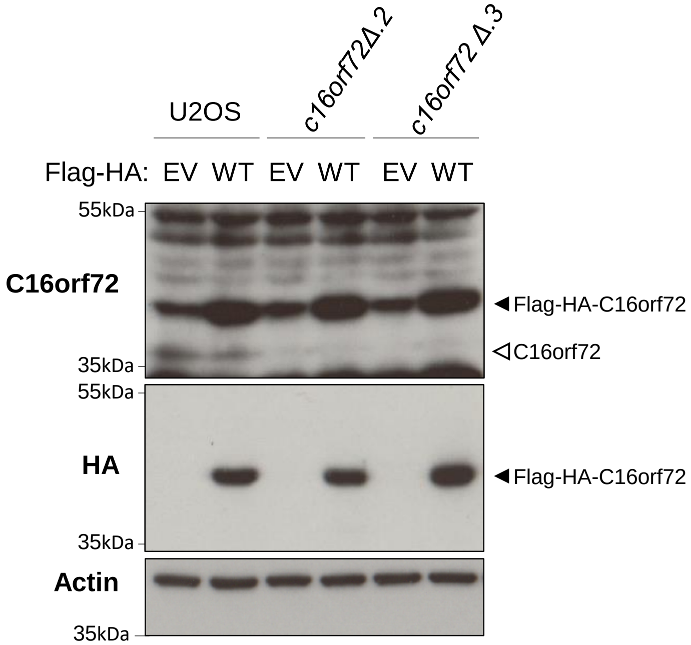

c

```
RPE1 WT          ATGGAGGAGCGGAAGGAGGAGGGCGAGGCCGAGATCCAGGAGCACGGGCCCGAGCACTGGTTCTCCAAGTGGGAGCGGCA
c16orf72ΔB10     ATGGAGGAGCGGAAGGAGGAGGGCGAGGCCGAGATCCAGGA---------(deletion of 56 bp)-----------
c16orf72ΔG5      ATGGAGGAGCGGAAGGAGGAGGGCGAGGCCGAGATCCAGGA---------(deletion of 56 bp)-----------
c16orf72ΔD4      ATGGAGGAGCGGAAGGAGGAGGGCGAGGCCGAGATCCAGGA---------(deletion of 164 bp)----------

RPE1 WT          GTGCCTGGCCGAGGCCGAACAGGACGAGCAGCTGCCCCCCGAGCTGCAGGAGGAGGCGGCGGCCGCCGCGCAGCCCGAGC
c16orf72ΔB10     -----------------AACAGGACGAGCAGCTGCCCCCCGAGCTGCAGGAGGAGGCGGCGGCCGCCGCGCAGCCCGAGC
c16orf72ΔG5      -----------------AACAGGACGAGCAGCTGCCCCCCGAGCTGCAGGAGGAGGCGGCGGCCGCCGCGCAGCCCGAGC
c16orf72ΔD4      --------------------------------------------------------------------------------

RPE1 WT          ACAAGCAGCAGAAGCTGTGGCACCTCTTCCAGAACTCGGCCACCGCCGTGGCCCAGCTCTACAAAGGTGAGGCCGCCGCC
c16orf72ΔB10     ACAAGCAGCAGAAGCTGTGGCACCTCTTCCAGAACTCGGCCACCGCCGTGGCCCAGCTCTACAAAGGTGAGGCCGCCGCC
c16orf72ΔG5      ACAAGCAGCAGAAGCTGTGGCACCTCTTCCAGAACTCGGCCACCGCCGTGGCCCAGCTCTACAAAGGTGAGGCCGCCGCC
c16orf72ΔD4      ----------------------------------------------CCGTGGCCCAGCTCTACAAAGGTGAGGCCGCCGCC
```

Supplementary Figure 1: Validation of *c16orf72Δ* cells. a. Genotype of U2OS *c16orf72Δ* cell lines. Genomic sequence of wildtype U2OS is given as reference; red: start codon; blue and green: CRISPR target sequences (PAM underlined). Clone *c16orf72Δ.2* is a compound heterozygote with one allele containing a single base insertion (white font in black background) and another allele with a 56-bp deletion. **b**. Western blot analysis of endogenous (top) and ectopically expressed Flag/HA-tagged C16orf72 protein (middle) in wildtype U2OS and *C16orf72* knock-out cells (*c16orf72Δ.2* and *c16orf72Δ.3*) transduced with lentivirus expressing Flag/HA tag (EV) or Flag/HA-C16orf72 (WT). Image is representative of 2 independent experiments. **c**. Genotype of RPE1 *c16orf72Δ* cell lines. Genomic sequence of wildtype RPE1 is given as reference; red: start codon; blue, green and orange: CRISPR target sequences (PAM underlined). Clones B10 and G5 were generated by targeting the blue and green sequences, whereas clone D4 was generated by targeting the blue and orange sequences.

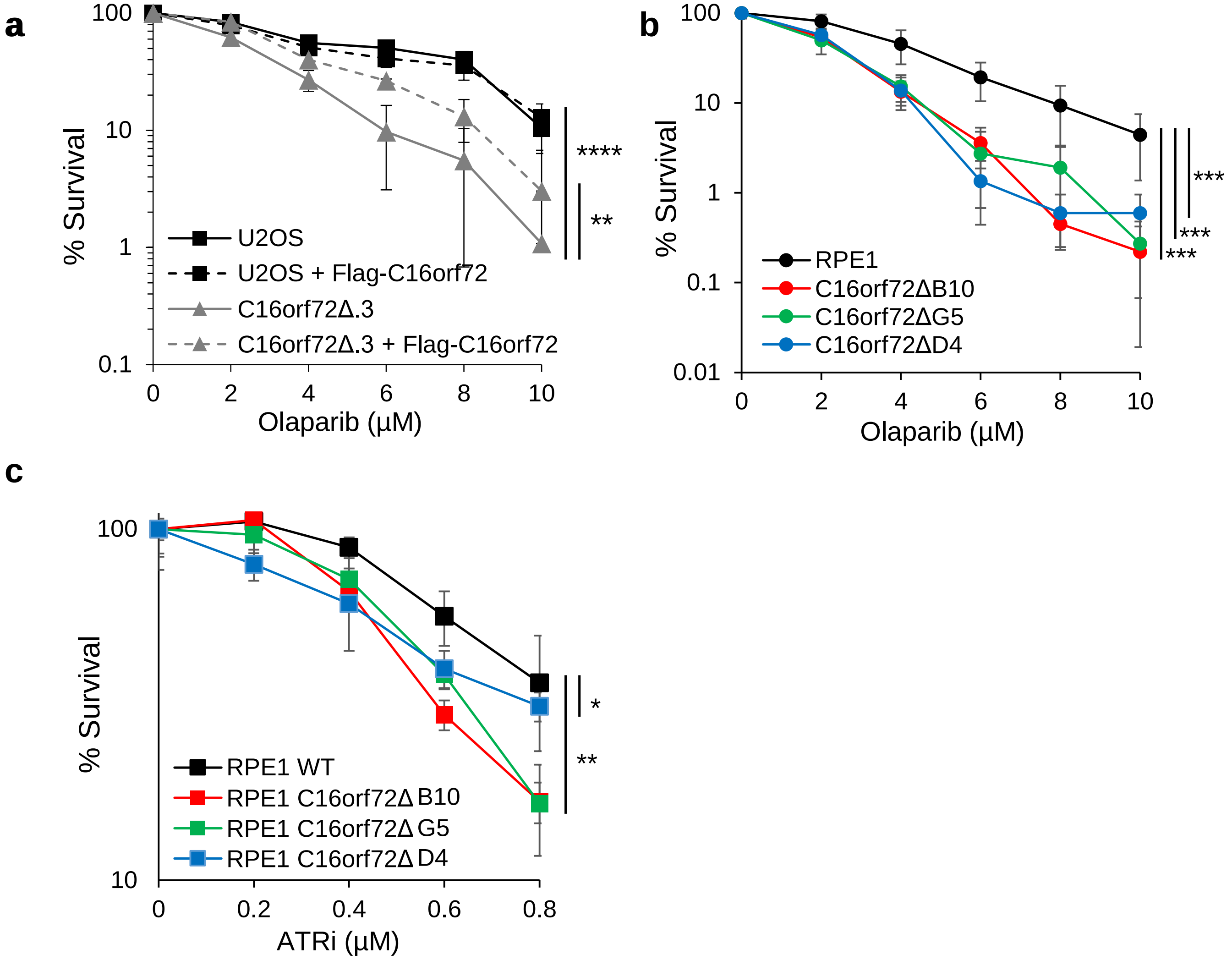

Supplementary Figure 2: Sensitivity of *c16orf72Δ* cells to PARP and ATR inhibitors. a. Clonogenic survival assay of *C16orf72* knock-out cells (*c16orf72Δ.3*) and complemented cells (*c16orf72Δ.3* cells ectopically expressing Flag-C16orf72) treated with increasing concentration of olaparib for 9 days. **b-c**. Clonogenic survival assay of *C16orf72* knock-out cells (*c16orf72ΔB10*, *c16orf72ΔG5* and *c16orf72ΔD4*) treated with increasing concentration of olaparib (b) or ATRi (c) for 9 days. All plots show the mean ± SEM where n= 3 biological independent experiments. Statistical analysis performed using two-way ANOVA with replication; * $p < 0.05$; ** $p < 0.01$; *** $p < 0.001$; **** $p < 0.0001$.

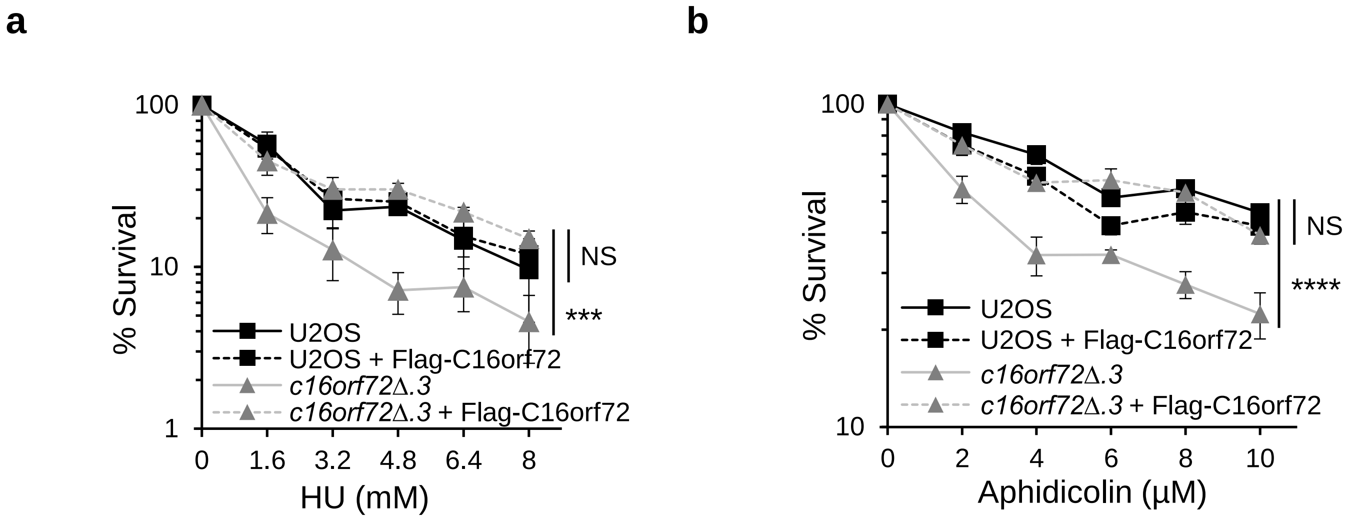

Supplementary Figure 3: Sensitivity of independent *c16orf72Δ* cells to agents that induce replication stress. a-b. Clonogenic survival assay of *C16orf72* knock-out cells (*c16orf72Δ.3*) and complemented cells (*c16orf72Δ.3* cells ectopically expressing Flag-C16orf72) treated with increasing concentration of hydroxyurea (a) or aphidicolin (b) for 24h. Plots show the mean ± SEM where n= 3 biological independent experiments. Statistical analysis performed using two-way ANOVA with replication; *** $p < 0.001$; **** $p < 0.0001$; NS: not significant.

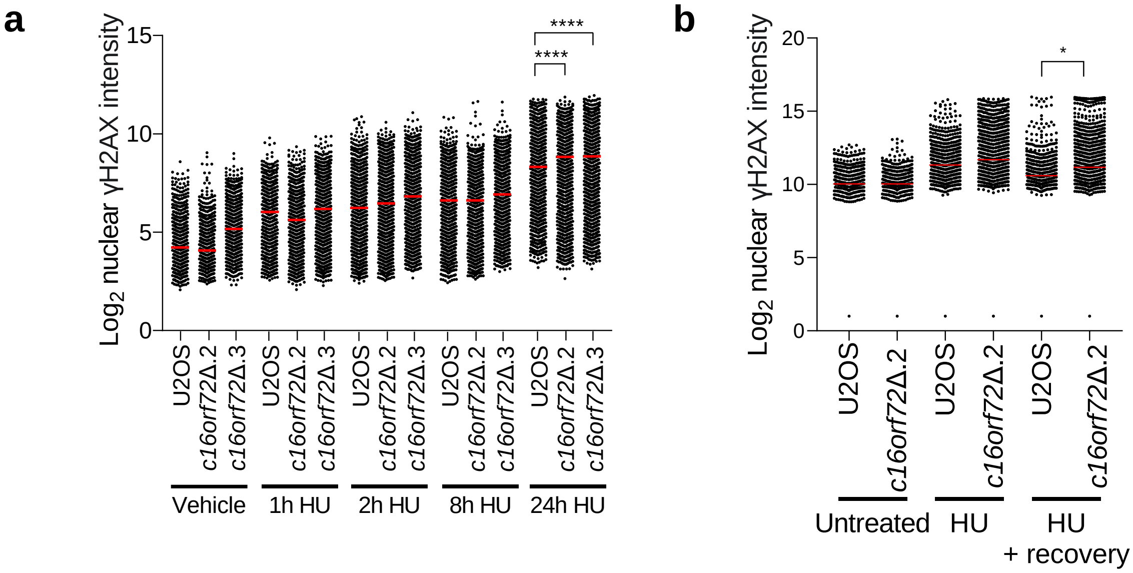

Supplementary Figure 4: Elevated and persistent levels of γH2AX following HU treatment of c16orf72 knockout cells

Quantitative image-based cytometry (QIBC) of data used in Figure 3a or Figure 4a representing induction of γ H2AX in response to HU (a) and decay following removal of the agent and 6 hours recovery (b), respectively. Individual cell data and mean nuclear intensity values (red lines) are plotted. Plots show the mean ± SEM where n= 3 biological independent experiments. Statistical analysis was performed using Ordinary one-way ANOVA with a Bonferroni post-hoc analysis; ns not significant, **** $p < 0.0001$ and *p= <0.01

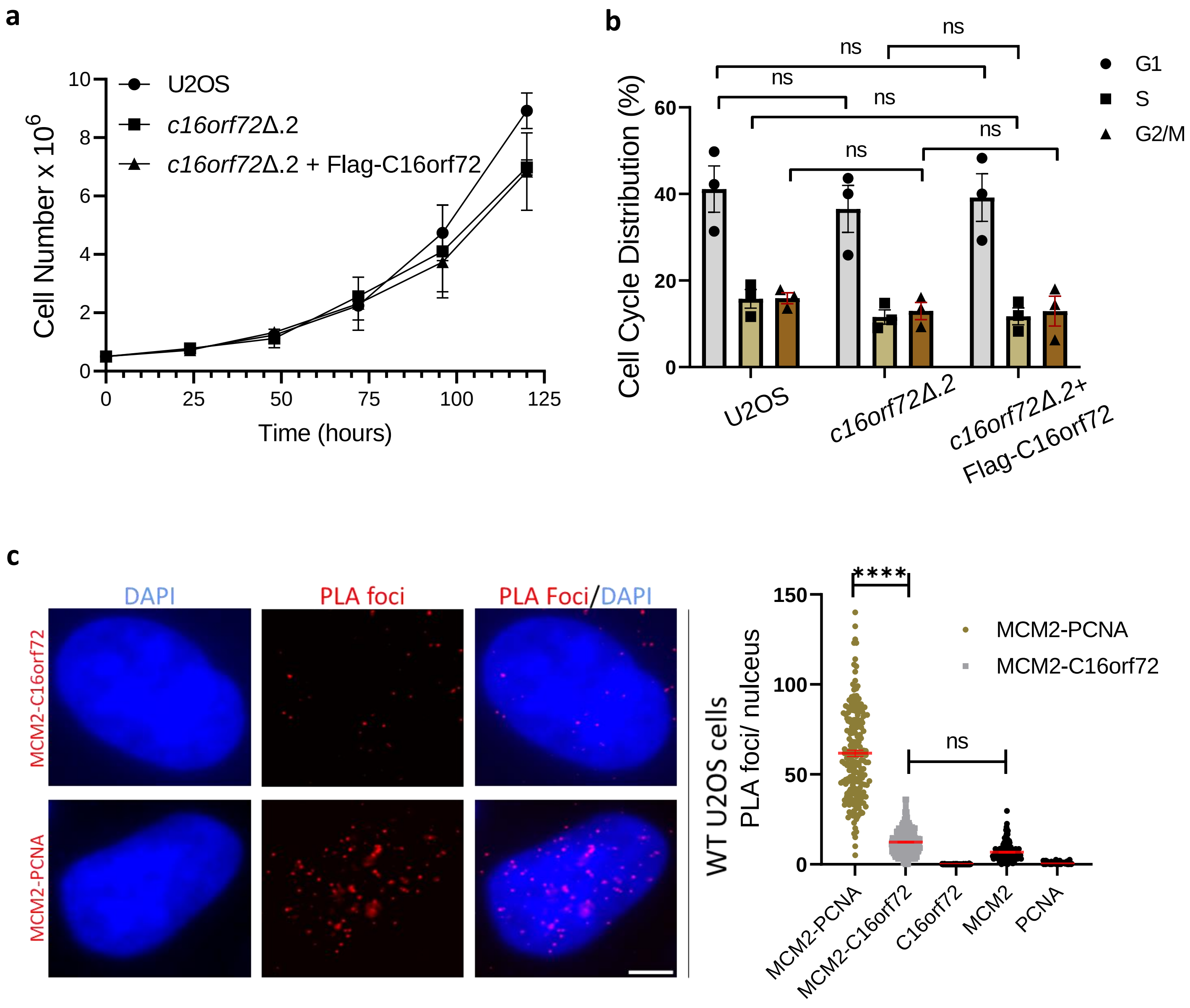

Supplementary Figure 5: C16orf72 status does not impact the cell cycle and C16orf72 is not a component of the replication fork. a. U2OS cells and *C16orf72* knock-out cells (*c16orf72*Δ.2) with or without Flag-C16orf72 expression were quantified for change in cell number after 24, 48, 96 and 120 hours. Data are presented as mean values and error bars represent +/- SEM of three independent experiments. **b**. U2OS cells and *C16orf72* knock-out cells (*c16orf72*Δ.2) with or without Flag-C16orf72 expression were subjected to cell cycle analysis by flow cytometry. Data are represented as the percentage of cells in different cell cycle phases and are presented as mean values with error bars representing +/- SEM of three independent experiments. **c**. U2OS cells were subjected to proximity ligation assays using the indicated antibody combinations. Representative images of PLA foci (red) and nuclei (DAPI) are illustrated. Scale bar=5µm. Individual data points are plotted along with mean values (red lines). Error bars represent +/- SEM of three independent experiments. Statistical significance was tested by one-way ANOVA or Kruskal Wallis non-parametric (****p ≤ 0.0001 and ns=non-significant)

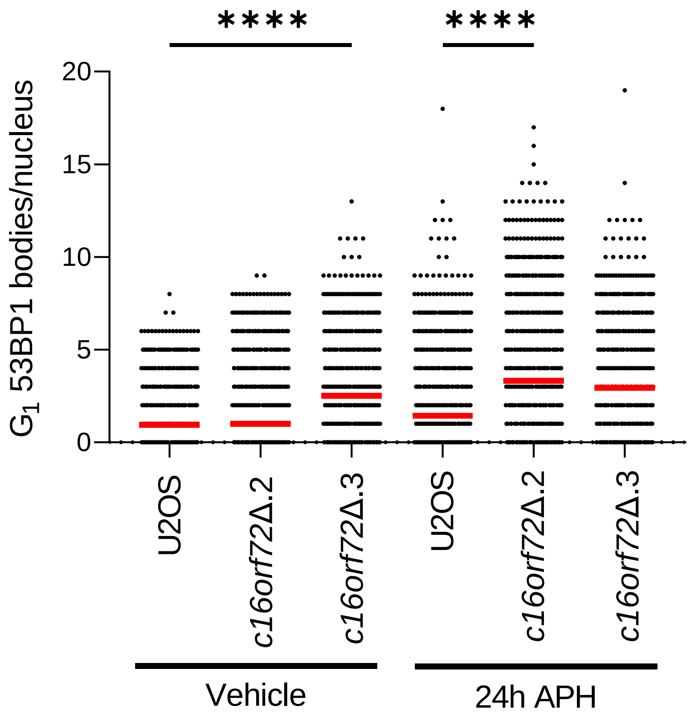

Supplementary Figure 6: Elevated 53BP1 bodies in G1 phase *c16orf72*∆ cells. Quantitative image-based cytometry (QIBC) of 53BP1 level in wild-type U2OS and C16orf72 knock-out cells left untreated or exposed to 0.2 μM aphidicolin for 24h. Data are those used in Figure 4e, although all data points are presented here. Mean 53BP1 bodies/nucleus are indicated (red lines) and error bars represent +/- SEM where n= 3 biological independent experiments. At least 400 G_1 cells (cyclin A-negative cells) were analysed per condition. For each condition, individual cell data is indicated (black dots). Statistical analysis was performed using Ordinary one-way ANOVA with Kruskal Wallis analysis; * $p < 0.0001$.

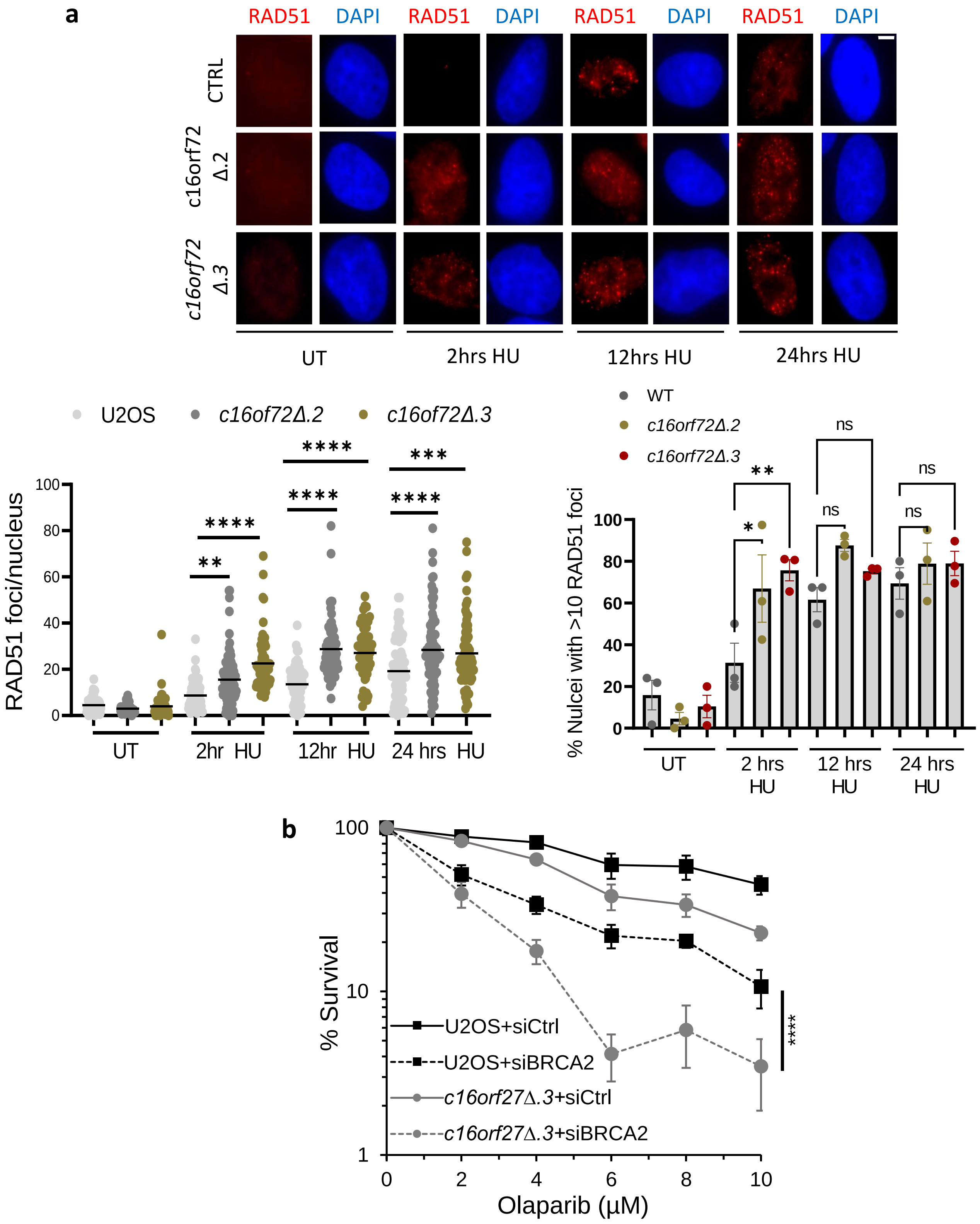

Supplementary Figure 7: C16orf72 does not function in the HR pathway to allow tolerance of cells to PARPi.

a. WT and *c16orf72Δ* U2OS cells were treated with 2mM HU for the indicated times (hr), subjected to IF using anti-Rad51 antibody and DAPI (top panel) and quantified for the Rad51 foci/nucleus (lower panel). Data is the mean Rad51 foci/nucleus (left panel) or mean % nuclei with >10 RAD51 foci (right panel) from the 40-87 cells per condition/independent repeat (n=3). Scale bar= 5µm. Error bars represent the ±SEM. Statistical significance was assessed by one-way ANOVA parametric or Kruskal Wallis non-parametric (*p ≤ 0.05, **p ≤ 0.01, *** p ≤ 0. 001, ****p ≤ 0.0001 and ns = non-significant) **b**. Clonogenic survival assay of wildtype U2OS and *c16orf72* knock-out cells (*c16orf72Δ.3*) transfected with siRNA targeting *BRCA2* or non-targeting control siRNA (siCtrl) treated with increasing concentration of olaparib for 48h. Mean ± SEM of 3 independent biological repeats. Statistical analysis performed using two-way ANOVA with replication; *** p < 0.001.

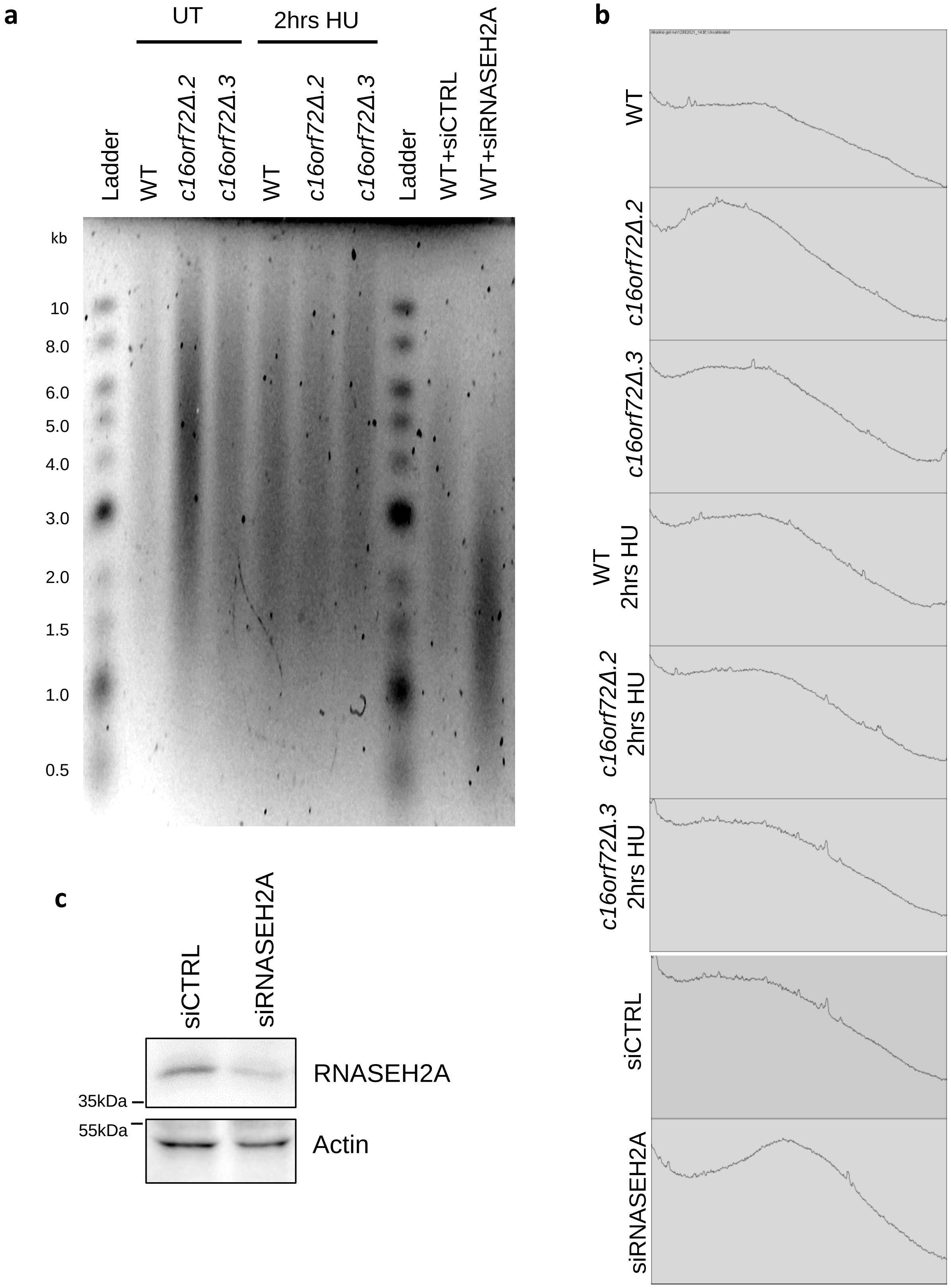

Supplementary Figure 8: C16orf72 does not function in ribonucleotide excision repair.
a. Wild-type U2OS (WT) or *c16orf72Δ* cells, or U2OS cells targeted with control or RNasH2A siRNA were left untreated or exposed to 2mM HU for 2 hours. Genomic DNA was isolated and 2.5μg DNA was incubated with 0.3M NaOH for 2.5 hours at 55°C and then subjected to low voltage alkaline gel electrophoresis, ethidium bromide staining and imaging. **b**. Quantification of grey value peaks of each sample from (a) corresponding to the shift in the genomic DNA migration upon alkaline treatment. **c**. Immunoblotting for RNASEH2A protein expression in the U2OS cells with indicated siRNA, probed with anti-RNASEH2A and anti-Actin antibody where Actin used as a loading control. Images in a and b are representative of 3 independent experiments.

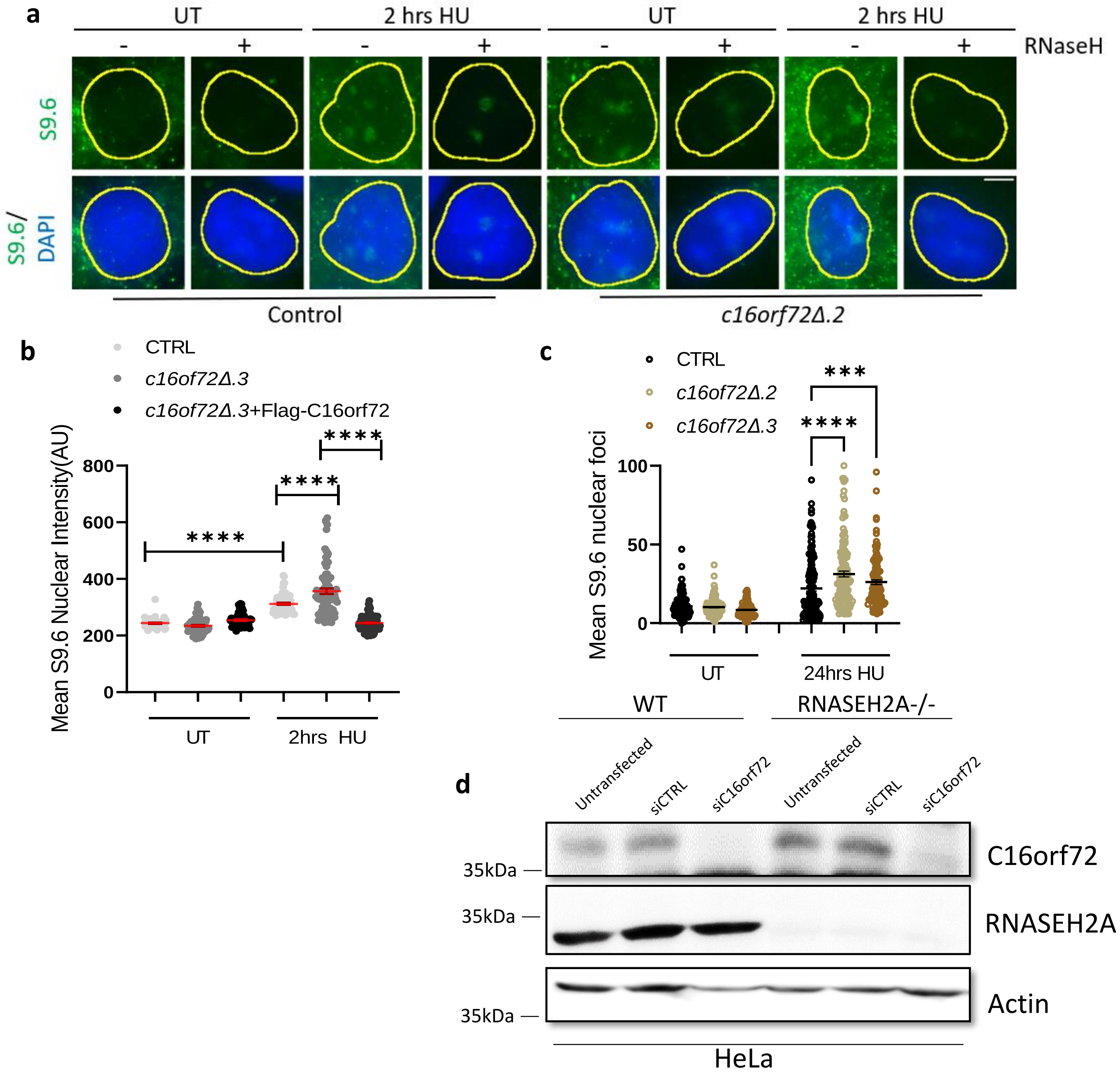

Supplementary Figure 9: Analysis of R-loops in *c16orf72*Δ cells and cells depleted for RNaseH2A. a. Representative images from the experiment in Figure 6d. WT U2OS (Control) and *c16orf72*Δ U2OS cells were treated with 2mM HU for the indicated times and subjected to either RNaseH treatment, or not, prior to immuno-fluorescence using S9.6 antibody to recognise DNA:RNA hybrids, or DAPI. The yellow boundary highlights the area of quantification (nucleus) for S9.6 intensity. Images are representative of 3 biological repeats and scale bar=5µm. **b**. Quantification of S9.6 mean nuclear intensity of WT U2OS (CTRL) and *c16orf72*Δ*.3* with or without expression of recombinant Flag-C16orf72 (c16orf72Δ.3+Flag-C16orf72). Mean values are represented (red lines) +/- SEM where n = at least 134 cells per treatment examined over 4 biological independent experiments, with exception of c16orf72Δ.3+Flag-C16orf72 (n=3 biological independent experiments). Statistical significance was tested by one-way ANOVA or Kruskal Wallis non-parametric (****p ≤ 0.0001). **c.** Data represent quantification of nuclear S9.6 foci subtracting the nucleolus foci. Mean values are represented (black lines) +/- SEM where n = at least 111 cells per treatment examined over 3 biological independent experiments. Statistical significance was tested by one-way ANOVA one-way ANOVA or Kruskal Wallis test (****p ≤ 0.0001 and *** p ≤ 0.001). **d**. RNASEH2A knock-out cells (RNASEH2A-/-), or parental HeLa cells were treated with control (siCTRL) or C16orf72 (siC16orf72) siRNA as indicated. Following preparation of whole cell extracts, western blotting was performed with the indicated antibodies to establish knock-down efficiency. Image is representative of 2 independent experiments.

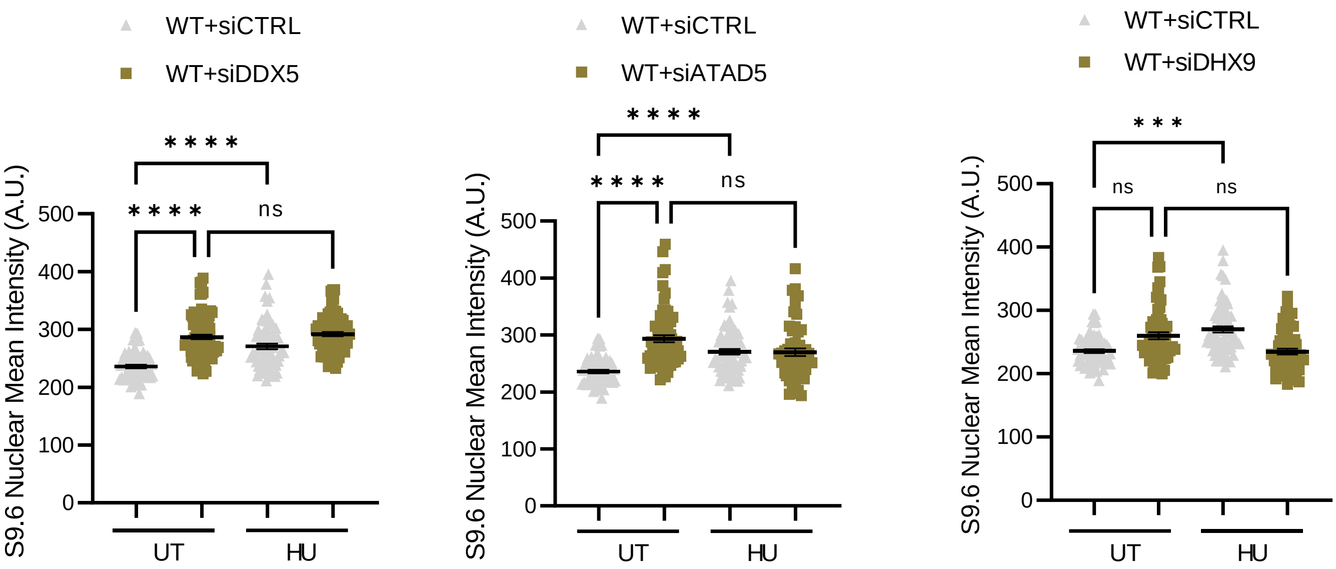

Supplementary Figure 10: Analysis of R-loops in U2OS cells with control and cells depleted for DDX5 or ATAD5 or DHX9 under replication stress.
U2OS cells were transfected with siControl (siCTRL) or with indicated siRNA for the knockdown of respective genes and later treated with 2mM HU for 2 hours and subjected to immuno-fluorescence using S9.6 antibody to recognise DNA:RNA hybrids and DAPI for nucleus. Graphs present the quantification of S9.6 nuclear mean intensity of U2OS with siCTRL and with siDDX5, siATAD5 and siDHX9. Mean values (black lines) are represented +/- SEM for at least 83 cells per treatment examined over 2 biological independent experiments. Statistical significance was tested by Kruskal Wallis non-parametric (***p≤0.001, ****p ≤ 0.0001 and ns=non significant).

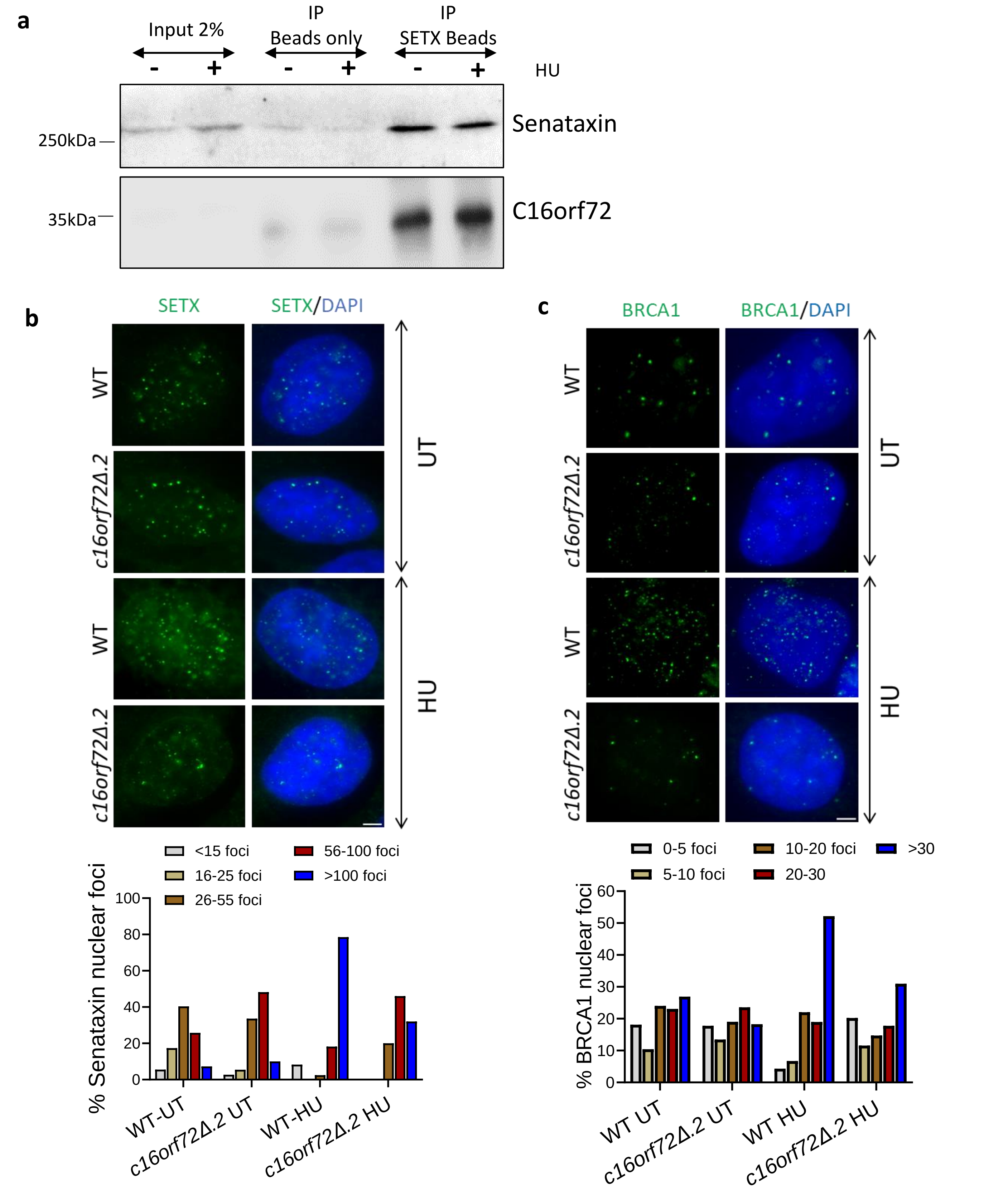

Supplementary Figure 11: Senataxin interacts with C16orf72 and is required to assemble BRCA1 and Senataxin into nuclear foci in response to replication stress. a U2OS cells were left untreated or exposed to 2mM HU for 2 hours. Following extract preparation, immunoprecipitations were performed with anti-senataxin coupled beads or beads alone as indicated. Immunoblotting was performed using the indicated antibodies. Images are representative of 3 biological repeats. **b. & c.** The indicated cell lines were incubated with/without 2mM HU for 2 hours and subjected to immunofluorescence for Senataxin and BRCA1. Images are the representative of the 3 biological repeats (above panel). Scale bar=5µM. Data presented in the bottom panels are the quantification of the % nuclei with different ranges of Senataxin (n>100 cells) and BRCA1 foci (n>180 cells) from the 3 biological repeats.
