# Supplementary material for: C16orf72/HAPSTR1/TAPR1 functions with BRCA1/Senataxin to modulate replication-associated R-loops and confer resistance to PARP disruption
Source: Nat Commun. 2023 Aug 17;14:5003. doi: 10.1038/s41467-023-40779-9 (PMC10435583; doi:10.1038/s41467-023-40779-9)
Supplement: Supplementary file 3 — Description of Additional Supplementary Files [file 41467_2023_40779_MOESM3_ESM.pdf]

Description of Additional Supplementary Files

File Name: Supplementary Data 1
Description: Genome-wide screen for synthetic lethal interactions with *parp1/2* gene disruption.
