# Supplementary material for: C16orf72/HAPSTR1/TAPR1 functions with BRCA1/Senataxin to modulate replication-associated R-loops and confer resistance to PARP disruption
Source: Nat Commun. 2023 Aug 17;14:5003. doi: 10.1038/s41467-023-40779-9 (PMC10435583; doi:10.1038/s41467-023-40779-9)
Supplement: Supplementary file 5 — Reporting Summary [file 41467_2023_40779_MOESM5_ESM.pdf]

nature portfolio

Corresponding author(s): Nicholas D. Lakin

Last updated by author(s): Aug 4, 2023

Reporting Summary

Nature Portfolio wishes to improve the reproducibility of the work that we publish. This form provides structure for consistency and transparency in reporting. For further information on Nature Portfolio policies, see our Editorial Policies and the Editorial Policy Checklist.

Statistics

For all statistical analyses, confirm that the following items are present in the figure legend, table legend, main text, or Methods section.

n/a	Confirmed	
☐	☒	The exact sample size (*n*) for each experimental group/condition, given as a discrete number and unit of measurement
☐	☒	A statement on whether measurements were taken from distinct samples or whether the same sample was measured repeatedly
☐	☒	The statistical test(s) used AND whether they are one- or two-sided<br>*Only common tests should be described solely by name; describe more complex techniques in the Methods section.*
☒	☐	A description of all covariates tested
☒	☐	A description of any assumptions or corrections, such as tests of normality and adjustment for multiple comparisons
☐	☒	A full description of the statistical parameters including central tendency (e.g. means) or other basic estimates (e.g. regression coefficient) AND variation (e.g. standard deviation) or associated estimates of uncertainty (e.g. confidence intervals)
☐	☒	For null hypothesis testing, the test statistic (e.g. *F*, *t*, *r*) with confidence intervals, effect sizes, degrees of freedom and *P* value noted<br>*Give P values as exact values whenever suitable.*
☒	☐	For Bayesian analysis, information on the choice of priors and Markov chain Monte Carlo settings
☒	☐	For hierarchical and complex designs, identification of the appropriate level for tests and full reporting of outcomes
☒	☐	Estimates of effect sizes (e.g. Cohen's *d*, Pearson's *r*), indicating how they were calculated

Our web collection on statistics for biologists contains articles on many of the points above.

Software and code

Policy information about availability of computer code

Data collection	IX71 Olympus confocal microscope using 60x and 100x objectives, Alpha Innotech Gel Imaging system, Liqor
Data analysis	ImageJ 1.53q, Microsoft Excel 2016, Prism Graphpad 9, MAGeCK 0.5.9

For manuscripts utilizing custom algorithms or software that are central to the research but not yet described in published literature, software must be made available to editors and reviewers. We strongly encourage code deposition in a community repository (e.g. GitHub). See the Nature Portfolio guidelines for submitting code & software for further information.

Data

Policy information about availability of data

All manuscripts must include a data availability statement. This statement should provide the following information, where applicable:

- Accession codes, unique identifiers, or web links for publicly available datasets
- A description of any restrictions on data availability
- For clinical datasets or third party data, please ensure that the statement adheres to our policy

All data generated or analysed during this study are included in this published article (and its supplementary information files), or available from the corresponding author on request.

Human research participants

Policy information about studies involving human research participants and Sex and Gender in Research.

Reporting on sex and gender	Not Applicable
Population characteristics	Not Applicable
Recruitment	Not Applicable
Ethics oversight	Not Applicable

Note that full information on the approval of the study protocol must also be provided in the manuscript.

Field-specific reporting

Please select the one below that is the best fit for your research. If you are not sure, read the appropriate sections before making your selection.

☒ Life sciences ☐ Behavioural & social sciences ☐ Ecological, evolutionary & environmental sciences

For a reference copy of the document with all sections, see nature.com/documents/nr-reporting-summary-flat.pdf

Life sciences study design

All studies must disclose on these points even when the disclosure is negative.

Sample size	As is standard practice for the field, all the experiments were concluded by independent biological repeats as specified in the Figure legends.
Data exclusions	No data was excluded from the analysis.
Replication	Data is derived from 2 to 5 independent biological experiments as specified in the Figure legends. All repeats were successful and where possible data used to perform statistical analysis.
Randomization	Samples were allocated into the group depending on cell treatments as described in the manuscript. Due to the relatively small group numbers randomization was not employed during these experiments
Blinding	Where possible (e.g. immunoflourescence), samples were scored blind. In certain experiments (e.g. western blotting) it was not possible to anonymise the data.

Reporting for specific materials, systems and methods

We require information from authors about some types of materials, experimental systems and methods used in many studies. Here, indicate whether each material, system or method listed is relevant to your study. If you are not sure if a list item applies to your research, read the appropriate section before selecting a response.

Materials & experimental systems

n/a	Involved in the study	
☐	☒	Antibodies
☐	☒	Eukaryotic cell lines
☒	☐	Palaeontology and archaeology
☒	☐	Animals and other organisms
☒	☐	Clinical data
☒	☐	Dual use research of concern

Methods

n/a	Involved in the study	
☒	☐	ChIP-seq
☒	☐	Flow cytometry
☒	☐	MRI-based neuroimaging

Antibodies

Antibodies used	HA (Cell Signaling Technology, 3724),<br>BRCA1 (D-9, SC6954, Santa Cruz Biotechnology),<br>BRCA2 (clone 5.23, 05-666 Millipore),<br>FANCD2 (Santa Cruz Biotechnology, sc-20022),<br>RNASEH2A(Santa Cruz Biotechnology, sc-515475),

HUWE1 (Bethyl Laboratories, A300-486A),
γH2AX for western blot (S319; Abcam, ab11174)
γH2AX for QIBC (Biolegend, 613402),
β-actin (Santa Cruz Biotechnology, sc-1615)
H3 (Abcam, ab12079),
phospho-RPA32 S4/S8 (Bethyl, A300-245A),
total RPA32 (Bethyl, A300-244A),
RPA70 (Abcam, ab79398),
phospho-DNA-PKcs S2056 (Abcam, ab124918),
total DNA-PKcs (Abcam, ab32566),
phospho-Chk1 S317 (Cell Signaling, D12H3, 12302),
total Chk1 (Cell Signaling, 2G1D5),
p53 (Santa Cruz, sc-126-HRP),
Vinculin (Santa Cruz, sc-73614-HRP),
53BP1 (Novus Biologicals, NB100-305),
Cyclin A (Santa Cruz, sc-751),
RAD51 (sc-8349, Santa Cruz Biotechnology),
Anti DNA-RNA Hybrid S9.6 clone (MABE1095, Millipore) and
Senataxin (QQ-7, sc-100319, Santa Cruz Biotechnology)
PCNA (D3H8P XP, Cell signalling)
MCM2 (Abnova 805-904)
The polyclonal antibody against the protein encoded by C16orf72 was raised in rabbit using a 16-residue peptide and was generated by Eurogentec.

Validation

HA (Cell Signaling Technology, 3724): https://www.cellsignal.co.uk/products/primary-antibodies/ha-tag-c29f4-rabbit-mab/3724
BRCA1 (D-9, SC6954, Santa Cruz Biotechnology): https://www.scbt.com/p/brca1-antibody-d-9
BRCA2 (clone 5.23, 05-666 Millipore): Refer to Cell, 104: 247-57 (2001) 2001
FANCD2 (Santa Cruz Biotechnology, sc-20022): https://www.scbt.com/p/fancd2-antibody-fi17
RNASEH2A(Santa Cruz Biotechnology, sc-515475): https://www.scbt.com/p/rnase-hii-a-antibody-g-10
HUWE1 (Bethyl Laboratories, A300-486A): https://www.thermofisher.com/antibody/product/Lasu1-Ureb1-Antibody-Polyclonal/A300-486A
γH2AX for western blot (S319; Abcam, ab11174): https://www.abcam.com/gamma-h2ax-phospho-s139-antibody-ab11174.html
γH2AX for QIBC (Biolegend, 613402): https://www.biolegend.com/en-us/products/purified-anti-h2a-x-phospho-ser139-antibody-1990?GroupID=GROUP26
β-actin (Santa Cruz Biotechnology, sc-1615): refer to Del Bello, B. et al. 2022. Cancer Cell Int. 22: 232
H3 (Abcam, ab12079): https://www.abcam.com/histone-h3-antibody-nuclear-marker-and-chip-grade-ab1791.html
phospho-RPA32 S4/S8 (Bethyl, A300-245A): https://www.thermofisher.com/antibody/product/Phospho-RPA32-Ser4-Ser8-Antibody-Polyclonal/A300-245A
RPA32 (Bethyl, A300-244A), https://www.thermofisher.com/antibody/product/RPA32-Antibody-Polyclonal/A300-244A-T
RPA70 (Abcam, ab79398), https://www.abcam.com/nav/primary-antibodies/rabbit-monoclonal-antibodies/rpa70-antibody-epr3472-ab79398.html
phospho-DNA-PKcs S2056 (Abcam, ab124918): https://www.abcam.com/dna-pkcs-phospho-s2056-antibody-epr5670-ab124918.html
total DNA-PKcs (Abcam, ab32566): https://www.abcam.com/dna-pkcs-antibody-y393-ab32566.html
phospho-Chk1 S317 (Cell Signaling, D12H3): https://www.cellsignal.co.uk/products/primary-antibodies/phospho-chk1-ser317-d12h3-xp-rabbit-mab/12302
total Chk1 (Cell Signaling, 2G1D5): refer to Garber, K. (2005) J Natl Cancer Inst 97, 1026-8
p53 (Santa Cruz, sc-126-HRP): https://www.scbt.com/p/p53-antibody-do-1
Vinculin (Santa Cruz, sc-73614-HRP): https://www.scbt.com/p/vinculin-antibody-7f9
53BP1 (Novus Biologicals, NB100-305): https://www.novusbio.com/products/53bp1-antibody_nb100-305
Cyclin A (Santa Cruz, sc-751): Sridhara, S.C. et al. 2017. Cell reports. 18: 334-343.
RAD51 (sc-8349, Santa Cruz Biotechnology): https://datasheets.scbt.com/sc-8349.pdf
Anti DNA-RNA Hybrid S9.6 clone (MABE1095, Millipore): https://www.merckmillipore.com/GB/en/product/Anti-DNA-RNA-Hybrid-Antibody-clone-S9.6,MM_NF-MABE1095
Senataxin (QQ-7, sc-100319, Santa Cruz Biotechnology): https://www.scbt.com/p/setx-antibody-qq-7
polyclonal antibody against the protein encoded by C16orf72 was raised in rabbit using a 16-residue peptide and was generated by Eurogentec.: The antibody was validated by Western Blot as presented in the manuscript, specifically by the band of predicted molecular weight being absent in c16orf72 KO cells (Supplementary Figure 1b)

Eukaryotic cell lines

Policy information about cell lines and Sex and Gender in Research

Cell line source(s)

All cell lines have been described previously (Ronson et al. (2018), Nat Commun 9:746), or are derived from U2OS cells used in this study. HeLa wild-type and RNASEH2A-null cells were a Gift from A Jackson (University of Edinburgh) and were described previously (Benitez-Guijarro M, et al. Embo J 37, [2018]). HEK293T cells used for virus production were from Lakin lab stocks.

Authentication

Cell lines were authenticated previously (Ronson et al. (2018), Nat Commun 9:746; Benitez-Guijarro M, et al. Embo J 37, (2018)). For derivatives of these cells generated during this study, the genotype was verified by PCR amplification and sequencing of the relevant loci and protein levels established by western blotting with the appropriate antibodies.

Mycoplasma contamination	All cell lines testes negative for mycoplasma infection
Commonly misidentified lines (See ICLAC register)	No commonly misidentified lines were used in this study.
