# Supplementary material for: C16orf72/HAPSTR1/TAPR1 functions with BRCA1/Senataxin to modulate replication-associated R-loops and confer resistance to PARP disruption
Source: Nat Commun. 2023 Aug 17;14:5003. doi: 10.1038/s41467-023-40779-9 (PMC10435583; doi:10.1038/s41467-023-40779-9)
Supplement: Supplementary file 6 — Source Data [file 41467_2023_40779_MOESM6_ESM.zip › P_values for Figures and Supplementary figures.docx]

P values for Figures

Figure 1b

siBRCA1: U2OS vs parp1/2Δ; p = 0.00918

siC16orf72: U2OS vs parp1/2Δ; p = 0.00502

Figure 1c

U2OS vs parp1/2Δ; p = 0.0023

Figure 1d

U2OS vs *c16orf72Δ.2*; p = 2.06E-06

*c16orf72Δ.2* vs *c16orf72Δ.2* + Flag-C16orf72; p = 2.06E-05

U2OS vs *c16orf72Δ.2* + Flag-C16orf72; p = 0.287

Figure 1e

*c16orf72Δ.2* vs *c16orf72Δ.2* + Flag-C16orf72; p=5.42E-6

Figure 2a

MMS:

U2OS vs *c16orf72Δ.2*; p = 0.443

U2OS vs *c16orf72Δ.3*; p = 0.292

U2OS vs parp1Δ; p = 1.80E-06

Phleomycin:

U2OS vs *c16orf72Δ.2*; p = 0.814

U2OS vs *c16orf72Δ.3*; p = 0.126

MMC:

U2OS vs *c16orf72Δ.2*; p = 3.79E-05

U2OS vs *c16orf72Δ.3*; p = 0.378

Figure 2b

U2OS vs *c16orf72Δ.2*; p = 1.74E-05

*c16orf72Δ.2* vs *c16orf72Δ.2* + Flag-C16orf72; p = 0.000158

U2OS vs *c16orf72Δ.2* + Flag-C16orf72; p = 0.0991

Figure 2c

U2OS vs *c16orf72Δ.2*; p = 2.54E-06

*c16orf72Δ.2* vs *c16orf72Δ.2* + Flag-C16orf72; p = 0.0529

Figure 3a

24h:

U2OS vs *c16orf72Δ.2*; p = <0.0001

U2OS vs *c16orf72Δ.3*; p = <0.0001

Figure 3b

24h, 2 mM HU:

U2OS vs *c16orf72Δ.2*; p = 0.001

U2OS vs *c16orf72Δ.3*; p = 0.01

Figure 4a

HU: U2OS vs *c16orf72Δ.2;* p=0.028

HU + 6h recovery: U2OS vs *c16orf72Δ.2*; p = 0.0061

Figure 4b

Untreated:

U2OS vs *c16orf72Δ.1*; p = <0.0001

*c16orf72Δ.1*vs *c16orf72Δ.1* + C16orf72; p = <0.0001

HU:

U2OS vs *c16orf72Δ.2*; p = <0.0001

*c16orf72Δ.2*vs *c16orf72Δ.2* + C16orf72; p = <0.0001

Figure 4c

Untreated:

U2OS vs *c16orf72Δ.1*; p = 0.002133918

U2OS vs *c16orf72Δ.1* + C16orf72; p = 0.095751083

U2OS vs *c16orf72Δ.2*; p = 0.000728554

U2OS vs *c16orf72Δ.2* + C16orf72; p = 0.319878469

HU:

U2OS vs *c16orf72Δ.1*; p = 0.001473282

U2OS vs *c16orf72Δ.1* + C16orf72; p = 0.119599682

U2OS vs *c16orf72Δ.2*; p = 0.004939196

U2OS vs *c16orf72Δ.2* + C16orf72; p = 0.624379769

Figure 4e

Untreated: U2OS vs *c16orf72Δ.3*; p = 0.0123

Aphidicolin: U2OS vs *c16orf72Δ.2*; p = 0.0223

Figure 5c

U2OS Untreated (UT) vs U2OS 2 hours (hrs) HU; p=<0.0001

Figure 5d

U2OS UT vs U2OS 2 hrs HU; <0.0001

U2OS 2 hrs HU vs *c16orf72Δ.2* 2hrs HU; p= <0.0001

U2OS 2 hrs HU vs *c16orf72Δ.3* 2 hrs HU; p=0.0013

U2OS 24 hrs HU vs *c16orf72Δ.2* 24 hrs HU; p=<0.0001

U2OS 24 hrs HU vs *c16orf72Δ.3* 24 hrs HU; p=0.0009

Figure 5e

U2OS UT vs U2OS 2 hrs HU; p=<0.0001

U2OS 2 hrs HU vs *c16orf72Δ.2* 2hrs HU; p=<0.0001

*c16orf72Δ.2* 2hrs HU vs *c16orf72Δ.2* 2hrs+Flag-C16orf72 2 hrs HU; p=<0.0001

Figure 5f

U2OS UT vs U2OS 2 hrs HU; p=<0.0001

U2OS 2 hrs HU vs *c16orf72Δ.2* 2hrs HU; p=<0.0001

U2OS 2 hrs HU vs *c16orf72Δ.3* 2hrs HU; p=<0.0023

*c16orf72Δ.2* 2hrs HU vs *c16orf72Δ.2* 2hrs HU+DRB; p=<0.0001

*c16orf72Δ.3* 2hrs HU vs *c16orf72Δ.3* 2hrs HU+DRB; p=<0.0001

Figure 6a

siRNASEH2A UT vs siRNASEH2A HU; p=<0.0001

siCTRL 2 hrs HU vs *c16orf72Δ.2* 2hrs; p=<0.0001

*c16orf72Δ.2* 2hrs vs *c16orf72Δ.2* +siRNASEH2A 2hrs; p=<0.0001

siRNASEH2A 2hrs vs *c16orf72Δ.2* +siRNASEH2A 2hrs; p=<0.0001

Figure 6b

siCTRL UT vs siCTRL 2 hrs HU; p=0.0022

siCTRL 2 hrs HU vs siC16orf72 2 hrs HU; p=<0.0001

siC16orf72 UT vs RNASEH2A-/- siC16orf72 UT; p=<0.0001

siC16orf72 2 hrs HU vs RNASEH2A-/- siC16orf72 UT 2 hrs HU; p=<0.0001

RNASEH2A-/- UT vs RNASEH2A-/- 2 hrs HU; p=<0.0001

RNASEH2A-/- 2 hrs HU vs RNASEH2A-/- siC16orf72 UT 2 hrs HU; p=<0.0001

Figure 6c

siCTRL UT vs siCTRL 2 hrs HU; p=<0.0001

siCTRL 2 hrs HU vs *c16orf72Δ.2* 2hrs; p=<0.0001

siCTRL 2 hrs HU vs siSETX 2 hrs HU; p=<0.0001

siCTRL 2 hrs HU vs *c16orf72Δ.2* +siSETX 2 hrs HU; p=<0.0001

Figure 6d

siCTRL UT vs siCTRL 2 hrs HU; p=<0.0001

siCTRL 2 hrs HU vs *c16orf72Δ.2* 2hrs; p=<0.0001

siCTRL 2 hrs HU vs siBRCA1 2 hrs HU; p=<0.0001

siCTRL 2 hrs HU vs *c16orf72Δ.2* +siBRCA1 2 hrs HU; p=<0.0001

Figure 6f

WT UT vs WT 2 hrs HU; p=<0.0001

*c16orf72Δ.2* UT vs *c16orf72Δ.2* 2 hrs HU; p=0.0076

WT 2 hrs HU vs *c16orf72Δ.2* 2 hrs HU; p=0.0026

Figure 6g

WT UT vs WT 2 hrs HU; p=<0.0001

WT 2 hrs HU vs *c16orf72Δ.2* 2 hrs HU; p=<0.0001

Figure 7a

WT+siCTRL vs WT+siBRCA1; p=0.0097

WT+siCTRL vs WT+siSETX; p=0.0015

WT+siCTRL vs *c16orf72Δ.2* +siSETX; p=0.0049

Figure 7b

WT+siCTRL vs WT+siSETX; p=<0.0001

WT+siCTRL vs *c16orf72Δ.2 +*siSETX; p=<0.0001

Figure 7c

WT+siCTRL vs WT+siBRCA1; p=0.0186

WT+siCTRL vs *c16orf72Δ.2* +siBRCA1; p=.0014

Figure 7d

WT UT vs WT Olaparib; p=<0.0001

*c16orf72Δ.2* UT vs *c16orf72Δ.2* Olaparib; p=<0.0001

WT Olaparib vs *c16orf72Δ.2* Olaparib; p=<0.0001

Figure 7e

WT+siCTRL vs WT+siBRCA1=<0.0001

WT+siCTRL vs *c16orf72Δ.2* +siCTRL*; p=<0.0001*

WT siBRCA1 vs *c16orf72Δ.2* +siCTRL p=<0.002

Figure 7f

WT+siCTRL vs *c16orf72Δ.2* +siCTRL; p=0.0034

WT+siCTRL vs WT+siSETX; p=<0.0001

Supplementary Data 2a

U2OS vs *c16orf72Δ.3*; p = 4.71E-09

*c16orf72Δ.3* vs *c16orf72Δ.3* + Flag-C16orf72; p = 0.00131

Supplementary Data 2b

RPE1 vs c16orf72ΔB10; p = 0.00159

RPE1 vs c16orf72ΔG5; p = 0.00396

RPE1 vs c16orf72ΔD4; p = 0.00242

Supplementary Data 2c

RPE1 vs c16orf72ΔB10; p = 0.00701

RPE1 vs c16orf72ΔG5; p = 0.0173

RPE1 vs c16orf72ΔD4; p = 0.000885

Supplementary Data 3a

U2OS vs *c16orf72Δ.3*; p = 0.000193

*c16orf72Δ.3* vs *c16orf72Δ.3* + Flag-C16orf72; p = 0.000124

U2OS vs *c16orf72Δ.3* + Flag-C16orf72; p = 0.532

Supplementary Data 3b

U2OS vs *c16orf72Δ.3*; p = 2.25E-08

*c16orf72Δ.3* vs *c16orf72Δ.3* + Flag-C16orf72; p = 9.08E-05

U2OS vs *c16orf72Δ.3* + Flag-C16orf72; p = 0.410

Supplementary Data 4a

24h:

U2OS vs *c16orf72Δ.2*; p = <0.0001

U2OS vs *c16orf72Δ.3*; p = <0.0001

Supplementary Data 4b

HU + recovery: U2OS vs U2OS vs *c16orf72Δ.2;* p=0.0055

Supplementary Data 5c

MCM2-PCNA vs MCM2-C16orf72; p=<0.0001

Supplementary Data 6

Vehicle U2OS vs Vehicle *c16orf72Δ.3; p=* <0.0001

24h APH U2OS vs 24h APH *c16orf72Δ.2; p=*<0.0001

Supplementary Data 7a

RAD51 foci/nucleus (left graph)

2hr HU:

U2OS vs *c16orf72Δ.2;* p=0.0021

U2OS vs *c16orf72Δ.3;* p=<0.0001

12hr HU:

U2OS vs *c16orf72Δ.2;* p=<0.0001

U2OS vs *c16orf72Δ.3;* p=<0.0001

24hrs:

U2OS vs *c16orf72Δ.2;* p=<0.0001

U2OS vs *c16orf72Δ.3;* p=0.0007

% Nuclei with >10 RAD%1 foci (right graph)

WT 2 hrs HU vs *c16orf72Δ.2* 2 hrs HU; p=0.0156

WT hrs HU vs *c16orf72Δ.3* 2hrs HU; p=0.0015

Supplementary Data 7b

U2OS + siBRCA2 vs *c16orf72Δ.3* + siBRCA2; p = 6.01E-06

Supplementary Data 9b

CTRL vs 2hrs HU CTRL; p=<0.0001

CTRL 2 hrs HU vs *c16orf72Δ.3* 2 hrs HU; p=<0.0001

*c16orf72Δ.3* 2 hrs HU vs *c16orf72Δ.3*+Flag+C16orf72 2 hrs HU; p=<0.0001

Supplementary Data 9c

CTRL 24hrs HU vs *c16orf72Δ.2* 24 hrs HU; p=<0.0001

CTRL 24hrs HU vs *c16orf72Δ.3* 24 hrs HU; p=<0.0002

Supplementary Data 10a

From the graph with siDDX5 data

WT siCTRL UT vs WT siDDX5 UT; p=<0.0001

WT siCTRL UT vs WT siCTRL HU; p=<0.0001

From the graph with siATAD5 data

WT siCTRL UT vs WT siATAD5 UT; p=<0.0001

WT siCTRL UT vs WT siCTRL HU; p=<0.0001

From the graph with siDHX9 data

WT siCTRL UT vs WT siCTRL HU; p=0.0010
